# The Role of Urocortins in Intracerebral Hemorrhage

**DOI:** 10.3390/biom10010096

**Published:** 2020-01-07

**Authors:** Ker Woon Choy, Andy Po-Yi Tsai, Peter Bor-Chian Lin, Meng-Yu Wu, Chihyi Lee, Aspalilah Alias, Cheng-Yoong Pang, Hock-Kean Liew

**Affiliations:** 1 Department of Anatomy, Faculty of Medicine, Universiti Teknologi MARA, Sungai Buloh 42300, Malaysia; celine_kerwoon@yahoo.com.tw; 2 Stark Neurosciences Research Institute, Indiana University School of Medicine, Indianapolis, IN 46202, USA; tandy@iu.edu (A.P.-Y.T.); pblin@iu.edu (P.B.-C.L.); 3 Department of Emergency Medicine, Taipei Tzu Chi Hospital, Buddhist Tzu Chi Medical Foundation, New Taipei 231, Taiwan; skyshangrila@gmail.com; 4 Department of Emergency Medicine, School of Medicine, Tzu Chi University, Hualien 970, Taiwan; 5 College of Pharmacy, University of Illinois at Chicago, Chicago, IL 60607, USA; chih.lee1227@gmail.com; 6 Department of Basic Sciences and Oral Biology, Faculty of Dentistry, Universiti Sains Islam Malaysia, Nilai 71800, Malaysia; draspa76@usim.edu.my; 7 Department of Medical Research, Hualien Tzu Chi Hospital, Buddhist Tzu Chi Medical Foundation, No. 707, Section 3, Zhong-yang Road, Hualien 970, Taiwan; 8 CardioVascular Research Center, Hualien Tzu Chi Hospital, Buddhist Tzu Chi Medical Foundation, Hualien 970, Taiwan; 9 Institute of Medical Sciences, Tzu Chi University, Hualien 970, Taiwan; 10 PhD Program in Pharmacology and Toxicology, Tzu Chi University, Hualien 970, Taiwan; 11 Neuro-Medical Scientific Center, Hualien Tzu Chi Hospital, Buddhist Tzu Chi Medical Foundation, Hualien 970, Taiwan

**Keywords:** corticotropin-releasing hormone receptor, endogenous neuropeptide, intracerebral hemorrhage, neuroprotective, urocortin

## Abstract

Intracerebral hemorrhage (ICH) causes an accumulation of blood in the brain parenchyma that disrupts the normal neurological function of the brain. Despite extensive clinical trials, no medical or surgical therapy has shown to be effective in managing ICH, resulting in a poor prognosis for the patients. Urocortin (UCN) is a 40-amino-acid endogenous neuropeptide that belongs to the corticotropin-releasing hormone (CRH) family. The effect of UCN is activated by binding to two G-protein coupled receptors, CRH-R1 and CRH-R2, which are expressed in brain neurons and glial cells in various brain regions. Current research has shown that UCN exerts neuroprotective effects in ICH models via anti-inflammatory effects, which generally reduced brain edema and reduced blood-brain barrier disruption. These effects gradually help in the improvement of the neurological outcome, and thus, UCN may be a potential therapeutic target in the treatment of ICH. This review summarizes the data published to date on the role of UCN in ICH and the possible protective mechanisms underlined.

## 1. Introduction

Intracranial hemorrhage is defined as bleeding in the brain parenchyma of the intracranial vault and meningeal spaces [1]. Intracerebral hemorrhage (ICH) that usually leads to disproportionately severe disability or death affects more than 1 million people every year [2]. The percentage of ICH is higher among Asians due to primary care limitations for uncontrolled hypertension and low compliance among patients [3]. Among Asians, the Han Chinese population has a higher incidence of ICH due to alcohol intake and hypertension [4].

Urocortin (UCN) is a peptide hormone that belongs to the corticotropin-releasing hormone (CRH) family of peptides [5]. UCN was found in the central nervous system and peripheral tissues [6]. The physiological function of UCN is mediated by CRH receptor 1 (CRH-R1) at the central nervous system and CRH-R2 at peripheral tissues [7]. UCN has been reported to possess various effects such as its ability to activate cellular metabolic pathways that control the functional central nervous system and the other system such as cardiovascular, gastrointestinal, reproductive and immune systems [8,9,10]. In vitro study has revealed that UCN possesses anti-inflammatory properties that reduced the neurotoxicity mediated by endotoxin-activated microglia. Intracerebroventricular treatment with UCN alleviated brain injury region, reduced edema of the brain, and improved blood-brain barrier (BBB) permeability, which generally improved neurological function in ICH animal study. UCN also decreases pathological changes in brain injury and neurological deficits in ICH rats.

The main objective of this article is to summarize the studies regarding the role of UCN with a focus on physiological and pathological conditions of ICH, which may potentiate a new strategy for clinical applications.

## 2. Intracerebral Hemorrhage

ICH, characterized by bleeding in the brain parenchyma, is a critical disease that could lead to severe morbidity and mortality worldwide [2,11]. ICH is divided into traumatic ICH and non-traumatic ICH, such as spontaneous or hypertensive ICH [11]. ICH can occur in different regions of the brain, at varying rates: the basal ganglia and internal capsule (35–70%), the brain stem (5–10%), the cerebellum (5–10%), or the cortical-subcortical areas (15–30%) [12,13]. The burden of ICH has been increasing in developing countries [14]. The severity and prognosis of ICH are different according to location, hematoma volume, size, and other factors [15].

### Epidemiology and Risk Factors

The Global Burden of Disease 2010 study demonstrated a 47% increase in cases of ICH between 1990 and 2010 worldwide [14]. In the United States, the incidence of ICH is around 40,000 cases per year [13]. ICH is more common in low- and middle-income countries, for example, southeast Asia, sub-Saharan Africa, and central Asia [14]. The fatality rate of IHC is high around 40% in the first month, and among these, only 12% to 39% of people will be able to survive with full functional independence [2].

Hypertension is the most common attributable risk factor for ICH [16]. Other modifiable risk factors accounted for ICH are low triglycerides, cigarette smoking, high alcohol consumption, diet, waist-to-hip ratio, reduced low-density lipoprotein cholesterol, and consumption of anti-coagulant or anti-thrombotic drug, respectively [17,18]. Moreover, being aging, male, or of Asian ethnicity, or having cerebral amyloid angiopathy or cerebral microbleeds are among the non-modifiable risk factors for ICH [19].

## 3. Molecular Pathology of ICH

**Figure 1:**
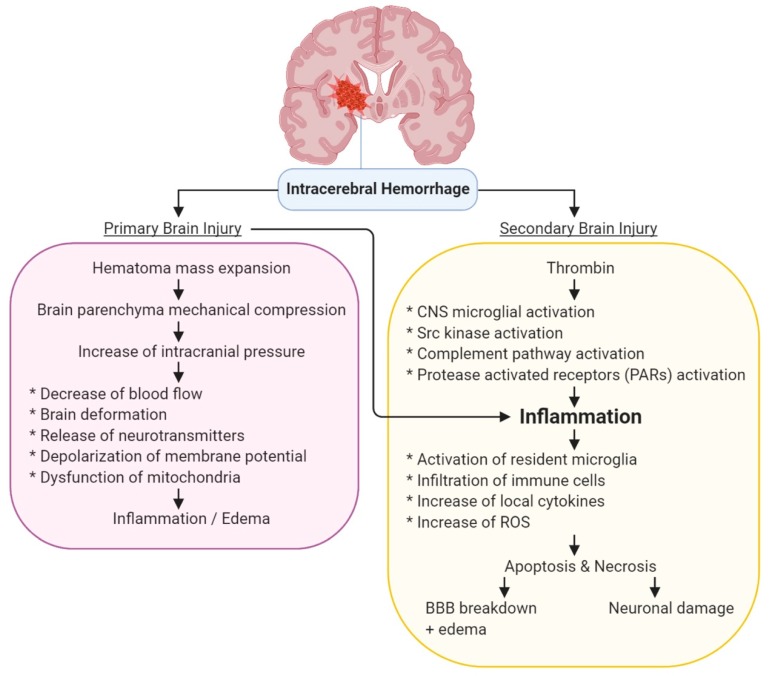
Primary and secondary injuries caused by intracranial hemorrhage. Abbreviations: ROS, reactive oxygen species; BBB, blood-brain barrier.

The pathogenesis of ICH consists of cumulative damages resulted from primary and secondary brain injury (Figure 1) [20].

### 3.1. Primary Injury by ICH

The primary mechanism of injury for ICH is caused by the physical destruction of parenchymal tissues by the hematoma mass expansion within 24 to 48 h after the injury [21]. The formation of hematoma causes mechanical compression to the brain parenchyma [22] that elevates intracranial pressure, affecting blood circulation, mechanical deformation, the release of neurotransmitters, depolarization of cell membrane, and dysfunction of mitochondria [23]. These events cause neuronal injury with edema and inflammation by blood-derived factors in the perihematomal area [23,24,25], which further associate to the deterioration of the neurological function of the brain as evidenced by changes in Glasgow Coma Scale and National Institutes of Health Stroke Scale scores [26].

### 3.2. Secondary Injury by ICH

The mechanism of the secondary injury for ICH could be triggered by primary injury (physical destruction of parenchymal tissues), tissue response to the hematoma (inflammation), or clotting factor release (hemoglobin or iron) [27]. The secondary injury occurs hours to days after the primary injury of ICH insult as the adjacent tissue responds to the endothelial damage and hemoglobin breakdown in the parenchyma near to the hematoma [28]. Blood components, such as red blood cells, leukocytes, macrophages, and plasma protein, infiltrate into the injury site and trigger an inflammatory response. The inflammation and tissue destruction in the hemorrhagic site includes inflammatory cell activation, activation of resident microglia, elevation of reactive oxygen species (ROS) [29], disruption of the blood-brain barrier (BBB), release of inflammatory mediators (e.g., interleukin-1β, which contributes to brain edema formation and cell necroptosis, and pro-inflammatory chemokines that amplify the existing pro-inflammatory mechanism), release of adhesion molecules, and release of matrix metalloproteinases (MMPs, especially MMP-9 and -3) that disrupt the BBB [28,30]. In addition, upon ICH, tumor necrosis factor-alpha (TNF-α) is activated, and consequently increases the release of proinflammatory cytokines, represses claudin-5 promoter activity through NF-κB signaling, upregulates excitotoxicity, and elevates the expression of vascular cell adhesion molecule 1 (VCAM-1) via phosphorylation of MAPKs on endovascular cells, respectively, leading to increases in the infiltration of leukocytes [30]. These inflammatory mediators also contribute to brain injury induced by surgical ICH in animals [28]. Disruption of BBB subsequently causes vasogenic edema formation in the affected hemisphere [31]. Each of these pathways could lead to the exacerbation of brain damage.

Binding of thrombin, a clotting cascade to protease-activated receptors 1, initiates central nervous system microglial activation and triggers various immune pathways, leading to apoptosis and necrosis of neurons and astrocytes [32]. High doses of thrombin infusion into the brain also leads to the infiltration of inflammatory cells, the proliferation of mesenchymal cells, scar formation, brain edema, and seizures [33]. Thrombin also initiates the activation of Src kinase, a protease-activated receptor, and the complement pathway, leading to mitogenic stress, excitotoxicity, inflammation, and vascular hyperpermeability [34,35]. Besides, upon endothelial damage, heme influx in neurons causes the release of iron and neuronal insult [36,37].

Since no medical or surgical therapy has shown to be effective in managing ICH, neutrophil-to-lymphocyte ratio (NLR) has been reported as a reliable measure of individual inflammatory status and predictor of clinical outcome [38]. An increased NLR represents the immune reactions triggered by cerebral hematoma and results in a prediction of hemorrhagic edema, early neurological deterioration, short-term mortality, and clinical outcome [39]. As well as NLR, increased C-reactive protein (CRP) has also been reported as a predication [40]. Therefore, serum inflammatory biomarkers have been proved as an outcome prediction of ICH through the role of inflammatory response.

## 4. General Management for ICH

ICH is a medical condition that requires urgent management as patients will experience a neurological decline, hematoma expansion, and a decrease in more than 2 points on the Glasgow Coma Scale (GCS) several hours post-injury [41]. The stabilization of airways, breathing, and circulation is vital at the initial stage of management for ICH patients [42]. When ICH is diagnosed, any antithrombotic agent such as vitamin K antagonists, direct factor Xa antagonists, direct thrombin inhibitors, unfractionated heparin, low-molecular-weight heparin, heparinoids, pentasaccharides, thrombolytics, and antiplatelet agents will be immediately discontinued [43]. As ICH is also associated with elevated systolic blood pressure, an intensive lowering of systolic blood pressure to <140 mm Hg has been shown to improve functional outcomes [44]. As well as the absolute blood pressure level, the increased variability of higher systolic blood pressure has been reported to be associated with the poor clinical outcome in ICH patients [45]. The study of INTERACT2 showed that patients with ICH would have the lowest risks when their systolic blood pressure could be controlled between 130 and 140 mm Hg [46,47]. For a patient with a hematoma of more than 30 cubic centimeters, mannitol or hypertonic saline (HTS) is prescribed to ensure serum sodium level at 140–150 mEq/L in order to reduce edema and elevated intracranial pressure, especially for a patient with a decreased level of consciousness [48]. If a patient manifested severe intraventricular hemorrhage with hydrocephalus, ventriculostomy will be performed [42]. As the physical disruption of the brain causes bleeding, increases intracranial pressure, and limits the blood flow of the affected brain region, patients with primary ICH may be indicated for surgery management such as a small craniotomy or corticectomy for hematoma evacuation [49].

The outcome of ICH is affected by several factors, such as low serum magnesium [50], inflammatory response (higher neutrophils and lower lymphocytes) [51], maintenance of cerebral perfusion pressure and drug actions [52,53] through the influence of neurovascular recovery and systemic complications. To identify future targets of ICH, biomarkers and systemic complication are needed to improve the prognostic algorithms. Although there are large numbers of clinical trials on ICH management, there is no medical or surgical procedure that shows a promising advantage for ICH patients [54]. Clinically, no drug has been proven to decrease the mortality rate of ICH patients [55]. Hyperosmolar therapy using HTS and/or mannitol for ICH with high intracranial pressure does not improve the outcome of the patients [48]. Hematoma evacuation using surgery has no significant advantage compared to conservative management [27,56], as the side effect of the surgery is greater than the benefit of the evacuation. Despite some survivors of ICH, the general prognosis of ICH is still poor, and the majority of the patients are left with a significant disability such as neglect, aphasia, loss of somatosensory, cognitive dysfunction, and depression [48]. Therefore, there is a need to look for other potential therapies for ICH, such as neuropeptide therapy.

## 5. Urocortin

### 5.1. Urocortin Structure, Expression, Signaling, and Receptors

Urocortin (UCN1 or UCN) belongs to the corticotropin-releasing factor (CRF) family of peptides, which include CRF, urocortin II (UCN2), urocortin III (UCN3), urotensin I, and sauvagine. UCN, first discovered in rat brains in 1995, is a 40-amino-acid peptide that has high sequence homology to urotensin (63% sequence identity) and CRF (45% sequence identity) [57]. UCN2 (also known as the stresscopin-related peptide) [58] and UCN3 (or stresscopin) [59] were identified later in 2001.

The presence of UCN differs in different regions of the brain. UCN’s location is mainly subcortical in the Edinger–Westphal nucleus [60], and its expression has also been documented in the pituitary gland [61]. In the peripheral tissues, UCN is located at tissue such as the adipose tissue [62], the heart [60], and cells involved in immunity including macrophages and fibroblasts [63], lymphocytes [64], and mastocytes [65].

UCN2 is present in the brain, but its function is largely unclear: UCN2 is found in the motor neurons of the brainstem, supraoptic nucleus, paraventricular nucleus of the hypothalamus, the arcuate nucleus and the locus coeruleus in the brainstem, and spinal cord [58]. In rats, UCN2 is isolated from the pituitary gland, the adrenal medulla, and the hypothalamus [66]. Besides, UCN2 was highly expressed in peripheral tissues such as the heart, lungs, adrenal glands, muscles, stomach, and blood cells [67].

UCN3 is mainly located in the amygdala, brainstem, and hypothalamus [59]. Its expression is also identified in other peripheral tissues such as heart and blood vessels [67], the skin [59], the adipose tissue [62], the thyroid gland, the pituitary gland, the spleen, the ovaries, the kidneys [68], beta cells of pancreas [69], the placenta and fetal membranes [70], and the muscularis mucosa of the gastrointestinal tract [71].

There are two related G protein-coupled CRF receptors, CRF receptor type 1 (CRFR1) and CRF receptor type 2 (CRFR2) [72]. UCN binds to both CRFR1 and CRFR2 [57], while UCN2 and UCN3 only react with CRFR2 [67,73]. After binding to its receptor, UCN will trigger two canonical pathways: (1) activation of phospholipase C and its downstream protein kinase C (PKC) pathway, and (2) activation of adenylyl cyclase and its downstream protein kinase A (PKA) pathway. These targeting pathways maintain cellular homeostasis and protect against cell injury. According to the protein structural study on human UCNs, UCN and UCN2 have two alpha-helical segments, while UCN3 only has one alpha-helix [74]. Despite there is a highly conserved structure across human and rat UCN (hUCN1: 51.22% alpha-helix; rUCN1: 51.22% alpha-helix), human UCN2 (hUCN2) and UCN3 (hUCN3) have lower similarity to the rat’s counterparts (hUCN2 and hUCN3: 58.97% alpha-helix; rUCN2 and rUCN3: 65.79% alpha-helix) [75]. 

The key determinant for activation of the CRF receptor is corticotropin-releasing factor-binding protein (CRF-BP), a 37 kD secreted glycoprotein [76]. The expression of CRH-BP is found throughout the brain, especially at parts such as the amygdala, hippocampus, ventral tegmental area, and prefrontal cortex [77]. CRF-BP of humans has a large affinity (picomolar range) to CRF and UCN of rats, as well as nanomolar affinity for UCN2 of mice [78]. However, human CRF-BP does not bind to hUCN2 and hUCN3 [78]. The binding of CRF-BP to CRF-family peptides is speculated to regulate the availability of the ligands to the two CRF receptors: CRFR1 and CRFR2 [59,79]. However, the actual physiological role of CRF-BP is still not fully elucidated. CRF-BP spontaneously cleavages into two smaller proteins, a 27 kD N-terminal that retains CRF-binding ability and a 10 kD C-terminal fragment of unknown function. Interestingly, the 10 kD CRF-BP fragment has been shown to potentiate the CRF-induced CRFR2 signaling when it was tethered to CRFR2 [76].

The activation of CRFR1 has been reported as a key factor that regulates emotional and cognitive outcomes, promoting the recovery of post-ischemic cognitive, depression and social impairment [80]. Through CRFR1, UCN mediates the neuroprotective effects of oxidative and excitotoxic cell death [81]. As UCN is a peptide involved in stress response, studies have suggested that the activation of CRF receptors might be detrimental, leading to chronic stress response and exacerbating neurodegeneration. Through the activation of cAMP response element-binding protein (CREB) in striatal neurons and hippocampal pyramidal neurons, UCN affects brain physiology via the stress response [82]. Also, UCN (CRFR1) is involved in stress-induced neurodegeneration, tau pathology, and cognitive performance [83]. Studies indicated that UCN and the activation of UCN receptors play an important role not only in the acute phase of stroke but also in chronic neurodegeneration. Therefore, more research is needed to develop a better strategy for UCN as a therapeutic agent in acute and chronic brain injuries.

### 5.2. Clinical Application of Urocortin in the Treatment of Heart Failure

UCN2 and UCN3 are expressed in the heart and may have beneficial effects on cardiovascular pathophysiology, particularly heart failure (HF) [84]. In a blind randomized crossover study, intra-arterial UCN2 and UCN3 were administrated using bilateral forearm venous occlusion plethysmography in healthy volunteers [85]. The results revealed that UCN2 and UCN3 significantly promote arterial vasodilatation without tachyphylaxis when compared to substance P (control) [85]. However, in patients with stable heart failure, there was no difference in terms of forearm vasodilatation within groups of patients infused with UCN 2 (3.6–36 pmol/min), UCN3 (360–3600 pmol/min) and substance P (2–8 pmol/min), while UCN3 caused a transient tachycardia at the highest dose infused, suggesting that vasodilator effects of UCN2 and UCN3 were upheld in patients with heart failure [86]. In contrast, other studies showed that intravenous delivery of UCN2 in HF patients increases their cardiac output and left ventricular ejection fraction while decreasing the systemic vascular resistance and cardiac work [87]. For acutely decompensated heart failure patients, UCN2 infusion leads to decreased systolic blood pressure, a reduction in total peripheral resistance, and increased cardiac output with a nonsignificant elevation in heart rate when compared to the placebo group [88]. David et al. (2005) evaluated the effect of UCN in stable congestive heart failure patients and found that UCN (50 μg) administration does not affect the cardiovascular system. They further suggested that the effects of UCN were not evident in heart failure in humans (Table 1) [89].

## 6. Urocortin and Intracerebral Hemorrhage

### 6.1. Urocortin Reduced Neurological Deficits

Stroke is one of the top risk factors that cause loss of neurological function and severe disability. Ten to twenty percent of strokes are caused by intracerebral hemorrhage. Symptoms in humans typically include a sudden and severe headache, dizziness, loss of balance, facial paralysis, hemiparesis, confusion, disorientation, and seizures [90]. Rodents are the most common model to understand human stroke since the loss of neurological function is related to the severity of the injury as well [91]. Identification of the behavioral deficits after stroke is important for potential translational applications. The modified neurological severity scores (mNSS) is a composite test of the motor, sensory, reflex and balance function, which rated the neurological function of rat (normal score 0; maximal deficit score 18) [29,92]. Recently, we demonstrated how rat UCN reduced neurological deficits caused by two different ICH rat models. In a dose-dependent manner, UCN alleviated neurological deficits in collagenase-induce ICH rats, while the lower 2.5 μg/kg UCN showed a greater reduction of neurological deficits compared to the 25 μg/kg UCN treated animals [20]. In another autologous blood infusion ICH model, UCN also alleviated neurological deficits in the ICH-insulted rats [20]. The results indicated UCN could serve as a potential therapeutic agent for ICH. Not only UCN, 2.5 μg/kg hUCN1 also reduced neurological deficits in ICH rats. The neurological deficits, however, did not mitigate when the rat was treated by hUCN2 and hUCN3 [75]. Neither hUCN2 nor hUCN3, which only bind to CRFR2, possessed a therapeutic effect on the neurological deficits of ICH rats, indicating CRFR1 might be a more specific target for hUCN1 in ICH.

### 6.2. Urocortin Has a Hypotensive Effect, but without Changing the Other Physiological Parameter

UCNs have been reported as clinically relevant molecules in the pathogenesis of hypertension, inflammatory disorders, depression, or obesity [5]. Most physiological parameters, such as blood pressure, heart rate, respiratory rate, and body temperature, were significant prognostic predictors in hemorrhagic stroke [93]. Chen et at. reported that hUCN2 induced hypotension through CRFR2 when given via intravenous injection [94]. Besides, UCN induced endothelium-dependent relaxation of rat coronary artery [95], rat thoracic aorta [96], renal arteries [97], and basilar artery [98], which indicated UCN might have a hypotensive effect. Different delivery routes of UCN, however, had a different outcome in the ICH rat model. Intraperitoneal injection of UCN (both 2.5 μg/kg and 25 μg/kg) decreased mean arterial blood pressure (MABP) and increased heart rate [20], but intracerebroventricular administration of UCN (5 μg per rat) did not cause a significant change of MABP. Physiological data also indicated acute blood pressure reduction might not play an important role in the cerebral ischemia of ICH patients [99]. Therefore, the MABP reduction might not become the concern of UCN treatment. Interestingly, different UCN treatments had various physiological effects, but most of the physiological parameters, such as pO2, pCO2, pH and body temperature, did not change [20,100].

### 6.3. Urocortin Reduced Brain Edema

The formation of brain edema can be classified into several phases: brain edema progressively increases after 24 h post-ICH, peaks approximately at 3 days post-ICH, and decreases after 9–14 days post-ICH. Clot retraction, inflammation, thrombin activation, erythrocyte lysis, and Hb toxicity-related injury were the reasons contributed to edema formation [101]. To date, approaches to brain edema reduction were limited clinically. UCN might be one of the potential treatments due to its effect on cerebral edema reduction. Post-treatment with UCN reduced cerebral edema of both contralateral and ipsilateral hemispheres on day 3 after collagenase-induced ICH in rats [20,100]. The results of UCN-reduced brain edema indicated UCN might solve the problem of short of effective treatment for ICH patients.

Preclinical studies showed that human corticotrophin-releasing factor (hCRF) possesses antiedema properties with less toxicity than dexamethasone (a standard drug for brain edema), and thus being proposed as a new treatment alternative for peritumoral brain edema [102,103]. Corticorelin acetate (Xerecept), a synthetic targeted human corticotrophin-releasing factor (hCRF) analog is a comparative drug that has been clinically investigated [104]. A clinical trial that involved 200 patients showed that hCRF is a well-tolerated drug as it is not associated with any significant side effect with single doses (1–5 μg/kg, i.v.) and continuous infusions of up to 2000 μg/24 h, i.y. per patients. Unless given at higher doses of up to 30 μg/kg, i.v., patients commonly developed hypotension, tachycardia, arrhythmias, and mental absences [104]. Other clinical trials showed that subcutaneous hCRF administrated to nearly 200 patients has long term safety and tolerability with reduced steroid-sparing effect in patients with primary or secondary brain tumors and peritumoral edema [105,106,107]. Similarly, a phase I randomized trial has demonstrated that hCRF injection is effective in relieving peritumoral brain edema, as well as its associated signs and symptoms, and thus, supports hCRF as a dexamethasone-sparing treatment for patients with symptomatic peritumoral brain edema (Table 2) [108].

### 6.4. Urocortin Reduced Pro-Inflammatory Cytokine Level in Striatal Tissue

Neuroinflammation is a hallmark in ICH that triggered by the endoplasmic reticulum (ER) stress and proteostasis disruption through early degradation of chaperone GRP78 and IkB protein, leading inflammatory response and increased cytokine expression [109]. Interestingly, a study has shown that the receptor of UCN, CRFR1, is involved in CRH-induced neuron apoptosis through the increased GRP78 in a concentration-dependent manner. From 10 nM to 10 µM, CRH decreased the cell viability from 28.9% to 42.9% of the control group [110]. ICH-induced proinflammatory cytokines, such as IL-1β, IL-6, and TNF-α, cause neuronal injury, result in apoptosis and necrosis [111], whereas UCN decreases ICH-induced level of pro-inflammatory cytokines in striatal tissue [20]. UCN also inhibits lipopolysaccharides (LPS)-induced tumor necrosis factor-α (TNF-α) in microglia through inhibition of phosphorylation of Akt and glycogen synthase kinase (GSK)-3β [112]. However, UCN inhibits TNF-α production from 1 fM, and the maximal effect was observed when microglia were pretreated with 10 nM UCN before LPS treatment. The results indicate that UCN concentrations are important for the therapeutic strategy.

Microglia and astrocytes provide nutritional support which maintains the physiological functions of neurons. UCN1 treatment attenuates ICH-related microglial activation-related neuronal loss [20]. These findings suggest that UCN1 mitigates pro-inflammatory cytokines release in striatal tissue via reducing microglial responses to ICH injury. The effects of UCN on ICH, however, have rarely been investigated. More and more studies are required to uncover the role of UCNs in ICH-mediated inflammatory response.

### 6.5. Urocortin Reduced Blood-Brain Barrier (BBB) Leakage

BBB, formed by cerebral endothelial cells, astrocytes, and pericytes, creates a barrier to separate peripheral circulation from the brain. Blood components derived from hematomas, such as thrombin, fibrin, and hemoglobin, triggered the disruption of BBB. ICH-induced cytokines also dysregulate the BBB function [113]. Evans blue binds to albumin, so, administration of Evans blue before sacrifice can be used to evaluate BBB integrity in the ICH animal model. UCN1 treatment decreased ICH-induced BBB dysfunction in the ipsilateral striatum and cortex [20]. UCN also attenuated hematoma volume, reduced pro-inflammatory cytokines release, and maintained the integrity of BBB after ICH injury. Xu et al. found similar results that UCN1 improved neurological deficits and reduced cerebral edema though CRFR2 by increasing vascular endothelial growth factor (VEGF) level. Also, the upregulated VEGF expression might enhance vascular formation, increasing oxygen and nutrients supply to the perihematomal tissues [114]. However, in the transient middle cerebral artery occlusion (tMCAO) mouse model, a study has shown that CRFR1 inhibition alleviated BBB disruption and neurovascular injury via the attenuation of cytosolic phospholipase A2 phosphorylation [115].

## 7. Conclusions & Perspective

The preclinical study of UCN had shown novel effects in the central nervous system, specifically in ICH. In the animal study, UCN has been proven as a potent anti-inflammatory agent that can improve neurological function, decrease the injured region, brain edema, and BBB permeability, likely via suppression of microglial activation and inflammatory cytokine production. It has also been shown that a low dose of UCN is more effective in reducing the functional deficits associated with ICH (Table 3).

However, in the clinical study, intra-arterial infusions showed that UCN2 and UCN3 do not show vasodilatation effects from low to high dosage. All the clinical studies are done in a small sample size. 

Nevertheless, UCN has great therapeutic potential as a target for the development of drug and clinical use. Further investigation with larger clinical sample size, a different formulation of UCN, and pre-clinical pharmacokinetic or pharmacodynamic study should be done to develop UCN as a therapeutic agent in ICH and other traumatic brain injuries.

## Figures and Tables

**Table 1:** Results of clinical trials from using urocortin as treatment for heart disease.

No	Intervention	Study Design	Results	Reference
1	UCN2, UCN3	Non-randomized clinical trial	Does not cause forearm vasodilatation in patients with stable heart failure. Highest dose of UCN3 triggers a transient tachycardia	[86]
2	UCN2	Single-blind, placebo-controlled, dose-escalation design	Increases cardiac output, elevates left ventricular ejection fraction, decreases systemic vascular resistance and cardiac work	[87]
3	UCN	Randomized time-matched cross-over design	No effect on cardiovascular system	[89]
4	UCN2	Randomized control trial	Reduces systolic blood pressure, decreases total peripheral resistance, elevates increased in cardiac output with nonsignificant elevation in heart rate	[88]

**Table 2:** Summary of clinical trials using hCRF for managing edema in patients with brain tumor.

No	Study Design	Results	Reference
1	A placebo-controlled study	hCRF improved symptoms of peritumoral edema associated with primary or metastatic cerebral tumors. hCRF enabled them to reduce or stop dexamethasone treatment and therefore minimize the rate of steroid-related short- and long-term adverse events of myopathy, cushingoid symptoms, and skin disorders	[105]
2	Randomized control trial	hCRF treatment had similar efficacy to increased dexamethasone. There was a lower incidence of cushingoid symptoms in the hCRF groups compared to dexamethasone groups	[106]
3	Randomized double-blind control trial	hCRF has long-term safety, tolerability, and reduced steroid-sparing potential in patients with primary or secondary brain tumors and peritumoral edema	[107]
4	Randomized control trial (Phase 1)	hCRF is well tolerated and effective in lowering peritumoral brain edema and its associated systemic side effects	[108]

**Table 3:** Results of urocortin in intracerebral hemorrhage.

	i.p.					i.c.v.		
	L-UCN (2.5 μg/kg)	H-UCN (25 μg/kg)	hUCN1 (2.5 g/kg)	hUCN2 (2.5 g/kg)	hUCN3 (2.5 g/kg)	UCN (0.05 g)	UCN (0.5 μg)	UCN (5 μg)
**Neurological deficits**								
NSS	↓↓	↓	↓	-	-	↓	↓	↓↓
Lesion volume	↓	N/A	↓	-	-	N/A	N/A	↓
**Physiological parameters**								
Mean arterial blood pressure (MABP)	↓	↓↓	N/A	N/A	N/A	N/A	N.A.	-
Heart rate	↑	↑	N/A	N/A	N/A	N/A	N/A	N/A
pO2	-	N/A	N/A	N/A	N/A	N/A	N/A	-
pCO2	-	N/A	N/A	N/A	N/A	N/A	N/A	-
pH	-	N/A	N/A	N/A	N/A	N/A	N/A	-
Body Temperature	-	N/A	N/A	N/A	N/A	N/A	N/A	-
**Brain edema**								
Water content	↓	↓	↓	N/A	N/A	N/A	N/A	↓
**Neuroinflammation**								
IL-1b	↓	N/A	N/A	N/A	N/A	N/A	N/A	N/A
IL-6	↓	N/A	N/A	N/A	N/A	N/A	N/A	N/A
TNF-a	↓	N/A	N/A	N/A	N/A	N/A	N/A	N/A
Microglial activation	↓	N/A	N/A	N/A	N/A	N/A	N/A	N/A
**BBB leakage**								
Evan’s blue	↓	N/A	↓	N/A	N/A	N/A	N/A	↓
Apparent diffusion coefficient (ADC)	N/A	N/A	↓	N/A	N/A	N/A	N/A	N/A
**References**	[20]		[75]			[100]		
